# Synthesis and evaluation in rats of homologous series of [^18^F]-labeled dopamine D_2/3_ receptor agonists based on the 2-aminomethylchroman scaffold as potential PET tracers

**DOI:** 10.1186/s13550-015-0119-x

**Published:** 2015-07-25

**Authors:** Vladimir Shalgunov, Jan-Peter van Wieringen, Henk M. Janssen, P. Michel Fransen, Rudi A.J.O. Dierckx, Martin C. Michel, Jan Booij, Philip H. Elsinga

**Affiliations:** Department of Nuclear Medicine and Molecular Imaging, University Medical Center Groningen, University of Groningen, Groningen, The Netherlands; Department of Nuclear Medicine, Academic Medical Center, University of Amsterdam, Amsterdam, The Netherlands; SyMO-Chem BV, Eindhoven, The Netherlands; Department of Pharmacology, Johannes Gutenberg University, Mainz, Germany

**Keywords:** Agonist tracer, Dopamine receptor, PET, Fluorine-18

## Abstract

**Background:**

Agonist positron emission tomography (PET) tracers for dopamine D_2/3_ receptors (D_2/3_Rs) offer greater sensitivity to changes in endogenous dopamine levels than D_2/3_R antagonist tracers. D_2/3_R agonist tracers currently available for clinical research are labeled with the short-lived isotope carbon-11, which limits their use. We aimed to develop high-affinity D_2_R agonists amenable for labeling with the longer-living fluorine-18. Here, we report the evaluation as potential PET tracers of two homologous series of [^18^F]fluorinated tracers based on the 2-aminomethylchroman-7-ol (AMC) scaffold: (R)-2-((4-(2-fluoroalkoxy)benzylamino)methyl)chroman-7-ols (AMC13 homologues) and (R)-2-((2-(4-(4-(fluoroalkoxy)phenyl)piperazin-1-yl)ethylamino)methyl)chroman-7-ols (AMC15 homologues). We varied the length of the ^18^F-fluoroalkyl chain in these structures to balance brain penetration and non-specific binding of the radioligands by adjusting their lipophilicity.

**Methods:**

The tracers were evaluated in brain slices of Sprague-Dawley rats by in vitro autoradiography and in living rats by microPET imaging and ex vivo autoradiography. PET data were analyzed with one- and two-tissue compartmental models (1TCM/2TCM), simplified reference tissue model (SRTM), and Logan graphical analysis. Specificity of binding was tested by blocking D_2/3_R with raclopride.

**Results:**

Homologues with a shorter fluoroalkyl chain consistently showed greater D_2/3_R-specific-to-total binding ratios in the striatum than those with longer chains. The fluoroethoxy homologue of AMC13 ([^18^F]FEt-AMC13) demonstrated the highest degree of D_2/3_R-specific binding among the evaluated tracers: mean striatum-to-cerebellum uptake ratio reached 4.4 in vitro and 2.1/2.8 in vivo/ex vivo (PET/autoradiography). Striatal binding potential (BP_ND_) relative to cerebellum was 0.51–0.63 depending on the estimation method. Radiometabolites of [^18^F]FEt-AMC13 did not enter the brain. In vitro, application of 10 μmol/L raclopride reduced D_2/3_R-specific binding of [^18^F]FEt-AMC13 in the striatum by 81 %. In vivo, pre-treatment with 1 mg/kg (2.9 μmol/kg) raclopride led to 17–39 % decrease in D_2/3_R-specific binding in the striatum.

**Conclusions:**

Varying the length of the [^18^F]fluoroalkyl chain helped improve the characteristics of the original candidate tracers. Further modifications of the current lead [^18^F]FEt-AMC13 can provide an agonist radiopharmaceutical suitable for D_2/3_R imaging by PET.

**Electronic supplementary material:**

The online version of this article (doi:10.1186/s13550-015-0119-x) contains supplementary material, which is available to authorized users.

## Background

Dysregulation of dopamine signaling through dopamine D_2_ and D_3_ receptors (D_2/3_Rs) is implicated in many neuropsychiatric disorders [1–4], making imaging of D_2/3_Rs by positron emission tomography (PET) highly relevant.

Majority of PET tracers developed for D_2/3_R imaging are antagonists [5], but in the last two decades, agonists have attracted attention as potential PET ligands for G protein-coupled neurotransmitter receptors (GPCRs). In in vitro competition studies, agonists consistently show higher affinity for receptor molecules bound to G proteins (“high-affinity state”) than for free receptors (“low-affinity state”) [6–8]. Agonists are therefore expected to selectively recognize the “high-affinity” subset of receptor population in the imaging experiments, while antagonists bind to all receptors disregarding “affinity states” [9].

Though the existence of a separate “high-affinity state” subpopulation of D_2/3_R in the brain is still not conclusively demonstrated in vivo [10], radiolabeled D_2/3_R agonists indeed turned out to be more sensitive than the antagonist [^11^C]raclopride to amphetamine-induced release of dopamine (itself also an agonist) in rodents, cats, non-human primates, and humans [11–17]. Alteration of the relative abundance of “high-affinity state” D_2/3_Rs (D_2/3_R-high), detected by in vitro methods, is implied in dopamine supersensitivity, a state relevant to psychosis, Parkinsonism, and drug addiction [18]. Changes in this in vitro-based parameter are likely to be translated into effects observable in vivo, so agonist tracers might provide new insights for the research into, and (early) diagnosis of, these common neuropsychiatric disorders.

Numerous scaffolds have been investigated in the development of D_2/3_R agonist PET tracers [19], but representatives of only two classes, namely the apomorphines [^11^C](−)NPA and [^11^C]MNPA and the naphtoxazine [^11^C](+)PHNO, have hitherto been used in human PET studies. These tracers can only be used in hospitals with an on-site cyclotron due to the short half-life of carbon-11. Fluorine-18-labeled tracers (half-life of 109.8 min) have greater potential of widespread application in the clinic and are also more suitable for long-duration experiments in the research set-up. [^18^F]5-OH-FPPAT, an aminotetraline tracer, showed promising results in rats and monkeys [20], but no evaluation in humans has been published so far. A [^18^F]fluorinated analog of [^11^C](+)PHNO failed to demonstrate D_2/3_R-specific binding in rats [21], while the recently described [^18^F]MCL-524, structurally related to [^11^C]MNPA, showed very promising results in non-human primates [22].

We aimed to increase the availability of fluorine-18-labeled D_2/3_R agonist tracers for PET. For this purpose, we selected the 2-aminomethylchroman-7-ol (AMC) scaffold. Structure-activity relationships of this class of high-affinity D_2_R-agonists were previously investigated by Mewshaw and co-workers [23].

**Fig. 1:**
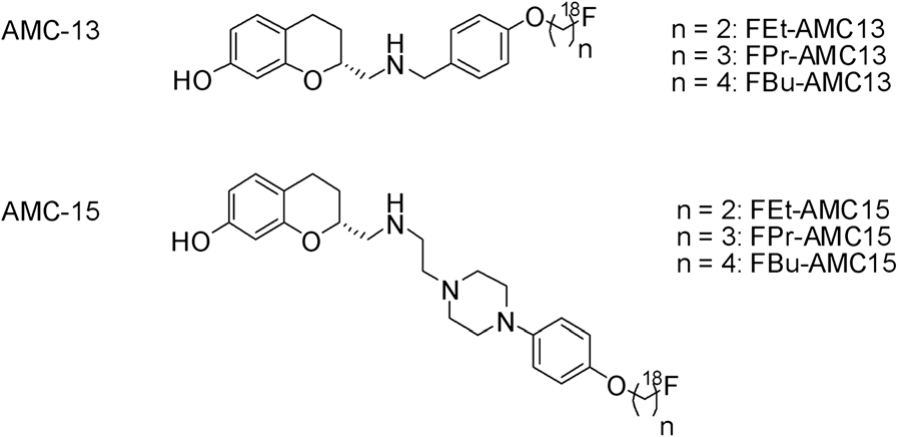
Structures of the evaluated homologues of AMC-13 and AMC-15

Recently, we have described the preparation of two [^18^F]-labeled AMCs (Fig. 1): (*R*)-2-[(4-(4-[^18^F]fluorobutoxy)benzylamino)methyl]chroman-7-ol ([^18^F]FBu-AMC13) and (*R*) 1-(4-(2-[^18^F]fluoroethoxy)phenyl)-4-(4-(7-hydroxychroman-2-yl)-3-azabutyl)-piperazine ([^18^F]FEt-AMC15). Both compounds, when tested in their non-radioactive [^19^F]-form, showed nearly full agonism at the long isoform of human D_2_R and low-nanomolar affinities towards the high-affinity state of these receptors: on average 5.6 and 6.7 nM for FBu-AMC13 and FEt-AMC15, respectively. The affinities of these two compounds towards human D_2_R and D_3_R were comparable to each other, with no detectable preference for any of the two receptor subtypes [24]. Both [^18^F]FBu-AMC13 and [^18^F]FEt-AMC15 also demonstrated specific binding to the D_2/3_R-high in rat striata. We wanted to further evaluate these radioligands in vivo and optimize their brain penetration and non-specific binding by adjusting their lipophilicity. We reasoned that the latter goal could be achieved by varying the length of the [^18^F]fluoroalkyl chain in the structures of the tracers, because these chains are situated outside the main pharmacophore of the AMCs [24], so shortening or elongating them will likely have no influence on the affinity of the resulting ligands towards D_2/3_R.

This study reports on the synthesis of [^18^F]-labeled AMC13 and AMC15 homologues with varied alkyl chain lengths, and on the in vitro and in vivo evaluation of these compounds.

## Methods

### Chemistry

Reagents, chemicals, materials, and solvents were obtained from commercial sources and were used as received: Biosolve, Merck for solvents, Cambridge Isotope Laboratories for deuterated solvents, and Aldrich, Acros, ABCR, Merck, and Fluka for chemicals, materials, and reagents. All solvents were of analytical grade quality.

^1^H-NMR and ^13^C-NMR spectra were recorded on Varian MR (300 or 400 MHz for ^1^H-NMR, 100 MHz for ^13^C-NMR) spectrometers at an ambient temperature. Chemical shifts are reported in ppm, applying deuterated chloroform (CDCl_3_) or other deuterated solvents as internal reference. Abbreviations used for splitting patterns are s = singlet, t = triplet, q = quartet, m = multiplet, and dd = double doublet.

LC-ESI-MS/MS analyses were performed on a Shimadzu LC-20AD series LC coupled to an API3000 mass spectrometer (Applied Biosystems/MDS SCIEX, Toronto, Canada), a triple quadrupole mass spectrometer supplied with an atmospheric pressure ionization source, and an ion-spray interface. Analyses were executed at 298 K using an Alltech Alltima C18 5 μ 150 × 2.1 mm column using an injection volume of 10 μL, a flow rate of 0.4 mL/min, and a gradient of MeCN/propan-2-ol in H_2_O (from 5 to 95 % organic component, where both MeCN/propan-2-ol and H_2_O contain 0.1 % formic acid). MS/MS analysis was performed in the multiple reaction monitoring mode.

Analytical thin layer chromatography (TLC) was performed on Kieselgel F-254 precoated silica plates. Normal-phase column chromatography was carried out on regular silica gel 60 (0.040–0.063, Merck).

1,2-Ethanediol ditosylate, 1,3-propanediol ditosylate, 1,4-butanediol ditosylate, 2-fluoroethyl tosylate, 3-fluoropropyl tosylate, and 4-fluorobutyl tosylate were prepared according to procedures described in the literature [24, 25]. Analytical data (^1^H-NMR, ^13^C-NMR) were according to expectations.

The preparation and characterization of phenol precursors for ^18^F-fluoroalkylation, (R) N-[7-(methoxymethoxy)chroman-2-yl]methyl 4-hydroxybenzyl amine, and (R) 1-(4-hydroxyphenyl)-4-(4-(7-hydroxychroman-2-yl)-3-azabutyl)-piperazine were described earlier by van Wieringen et al. [24].

[^19^F]-references for FBu-AMC13 and FEt-AMC15 were prepared and characterized as described previously by van Wieringen et al. [24]. Of the new homologous ligands, [^19^F]-reference was only prepared for FEt-AMC13, the homologue that showed the most promising results in vivo. Given the use of the very similar radiolabeling methods for all ligands, and the close structural relatedness of the new homologues to the originally developed FBu-AMC13 and FEt-AMC15, we decided not to prepare [^19^F]-references for other homologues.

#### (R)-2-[(4-(4-Fluoroethoxy)benzylamino)methyl]chroman-7-ol ([^19^F]-reference of FEt-AMC13)

A solution of (*R*) N-[7-(methoxymethoxy)chroman-2-yl]methyl 4-hydroxybenzyl amine (28.4 mg, 0.11 mmol) in 1.5 ml dry DMF and 5 mg NaH were stirred in an oil bath at 80 °C. To this mixture 2-fluoroethyl tosylate (22.8 mg, 0.11 mmol) dissolved in 1.2 ml dry DMF was added in three portions of 0.4 ml over the period of 1 hour. The mixture was stirred at 80 °C for 24 h. After that, the reaction mixture was cooled down and N_2_ was bubbled through the solution to create an inert atmosphere. Then, 2 mL of 4 M HCl solution in dioxane was added to the mixture, and it was stirred for 5 h at room temperature in the atmosphere of N_2_. N_2_ was further bubbled through the solution to remove volatile reaction products from the mixture. Thereafter, the reaction mixture was diluted with 50 mL water and the pH was brought to 8–8.5 by addition of a Na_2_CO_3_-solution. The mixture was extracted by CH_2_Cl_2_ (3 × 10 mL), organic extract was washed with water (3 × 10 mL), dried with Na_2_SO_4_, and evaporated in vacuo. The residue was purified using silica column chromatography (ethyl acetate/methanol/triethylamine 100/5/1), giving 8.5 mg of the product (24% yield), as a slightly yellowish solid.

^1^H NMR (CD_3_OD/CDCl_3_): *δ* = 7.37 (d, 2H), 6.98 (d, 2H), 6.87 (d, 1H), 6.39 (dd, 1H), 6.21 (d, 1H), 4.83 (t, 1H), 4.71 (t, 1H), 4.29 (t, 1H), 4.22 (t, 1H), 4.12 (m, 1H), 4.01 (bs 2H), 3.01 (m, 2H), 2.9–2.5 (multiple signals, 2H), 2.05–1.65 (multiple signals, 2H). ^13^C NMR (CD_3_OD/CDCl_3_): *δ* = 162.7, 160.0, 158.2, 134.5, 133.9, 133.0, 118.8, 116.6, 112.5, 107.0, 86.6, 84.8, 76.6, 71.3, 71.1, 55.6, 55.2, 35.7, 33.4, 29.5, 27.3.

### Radiochemistry

**Fig. 2:**
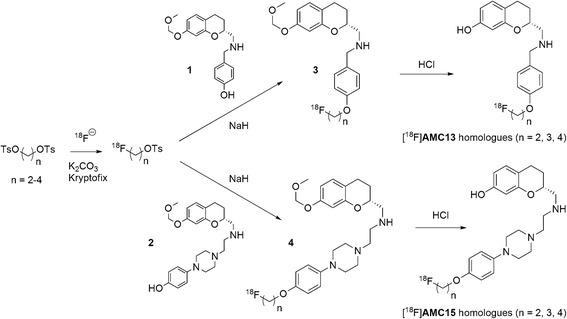
Labeling routes for the homologous series of [^18^F]AMC13 and [^18^F]AMC15

[^18^F]-fluoride was produced in the Scanditronix MC-17F cyclotron (GE Healthcare) and delivered from the target into the hotcell with helium overpressure. Fig. 2 gives an overview of the applied radiochemistry.

Synthetic procedures were performed manually. Radiochemical conversions and purities were determined by radio-HPLC using beta-sensitive detectors and dose-calibrators (Veenstra Instruments) and by radio-TLC using Cyclone phosphor storage screens (multisensitive, Packard) and OptiQuant software.

#### Preparation of [^18^F]fluoroalkyl tosylates

[^18^F]-Fluoride in enriched H_2_^18^O water was adsorbed on an activated anion-exchange column (QMA) and later eluted into the reaction vial with 1–1.5 mL of 1.5 mg/mL K_2_CO_3_, 5 mg/mL Kryptofix solution in 9:1 *v*/*v* mixture of acetonitrile and water, respectively. After the elution of QMA, acetonitrile and water were evaporated at 130 °C under nitrogen flow. For further water removal, 1 mL anhydrous acetonitrile was added into the reaction vial and evaporated. Then, 5 mg of alkyl ditosylate precursor (ethyl, propyl, or butyl) in 1 mL anhydrous acetonitrile was added into the vial, the vial was sealed, and reaction was carried out for 4 min at 130 °C followed by cooling 5 min. To the cooled reaction mixture, 1 mL of water was added, and the resulting mixture was loaded into the HPLC loop. [^18^F]fluoroalkyl tosylates were purified by reverse-phase HPLC using Alltima C18 5μ 250 × 10 mm column eluted with acetonitrile/water at 5 mL/min. Acetonitrile content in the mobile phase was 65 % for [^18^F]fluorobutyl tosylate and 50 % for [^18^F]fluoropropyl and [^18^F]fluoroethyl tosylate. Under these conditions, retention times of all thee [^18^F]fluoroalkyl tosylates were in the range of 6–8 min. Collected radioactive fraction was diluted two- to threefold with water and 4-[^18^F]fluoroalkyl tosylate was adsorbed on tC18 light Sep-Pak cartridge. The cartridge was rinsed with 5 mL pure water and dried for 5 min in strong nitrogen flow.

#### Preparation of [^18^F]FEt-AMC13, [^18^F]FPr-AMC13, and [^18^F]FBu-AMC13

(R) *N*-[7-(Methoxymethoxy)chroman-2-yl]methyl 4-hydroxybenzyl amine (1, 0.6 mg) in 30 μL dry DMF and 2–3 mg NaH were put into a V-vial. Thereafter, the corresponding [^18^F]fluoroalkyltosylate was eluted into the vial from the tC18 light Sep-Pak cartridge with 0.6–0.7 mL dry DMF. The reaction vial was sealed and the alkylation reaction to acquire compounds 3 were conducted for 10 min at 120 °C. Then, 1 mL 0.4 N HCl was added into the vial with a syringe and deprotection of the MOM-group followed that also lasted 10 min at 120 °C. The product was purified by reverse-phase HPLC using Alltima C18 5μ 250 × 10 mm column eluted with acetonitrile/10 mM H_3_PO_4_ at 5 mL/min. Acetonitrile content in the mobile phase was 30 % for [^18^F]FEt-AMC13 and 35 % for [^18^F]FPr-AMC13 and [^18^F]FBu-AMC13. Retention times were 10.6 min for [^18^F]FEt-AMC13, 9.2 min for [^18^F]FPr-AMC13, and 10.8 min for [^18^F]FBu-AMC13. Collected HPLC fraction was diluted with 2–3 volumes of water and the radioactivity was adsorbed on Oasis HLB SPE cartridge. The cartridge was rinsed with 5 mL pure water and radioactivity was then eluted with 1–2 mL absolute ethanol. Typical recovery was 20–40 % for pure ethanol and 80–90 % for 100/1 *v*/*v* mixture of ethanol with acetic or ortho-phosphoric acid; in synthesis runs for in vivo experiments, only pure ethanol was used. After partial or complete evaporation of ethanol in nitrogen flow (with or without application of vacuum), the tracer was formulated in physiological saline (ethanol content in the formulated tracer was kept below 10 %).

#### Preparation of [^18^F]FEt-AMC15, [^18^F]FPr-AMC15, and [^18^F]FBu-AMC15

The procedure described above for [^18^F]AMC13 homologues was also used for the preparation of [^18^F]AMC15 homologues, with the following modifications:

Instead of (R) *N*-[7-(methoxymethoxy)chroman-2-yl]methyl 4-hydroxybenzyl amine (1), (R) 1-(4-hydroxyphenyl)-4-(4-(7-hydroxychroman-2-yl)-3-azabutyl)-piperazine (2) was used as the precursor. MOM-protected molecules 4 were the intermediates for the [^18^F]AMC15 homologues (Fig. 2). At the end of the deprotection step, the reaction mixture was diluted with 10 mL water, titrated with 1 M NaOH to pH of 4–7 and then put through the Oasis HLB SPE cartridge. Adsorbed activity was then eluted from the cartridge with 1 mL acetonitrile/DMF mixture (1/1 *v*/*v*). Eluate was further diluted with water to a total volume of 1.5–2 mL, and the crude product was purified by HPLC using Alltima C18 5μ 250 × 10 mm column. For [^18^F]FEt-AMC15 purification, the column was eluted with a gradient of acetonitrile in 10 mM acetate buffer with pH 4.0 at 5 mL/min (45 % acetonitrile during the first 2 min, then a linear decrease from 45 % at 2 min to 30 % acetonitrile at 15 min). Retention time of [^18^F]FEt-AMC15 was 8.4 min. [^18^F]FPr-AMC15 and [^18^F]FBu-AMC15 purification the column was eluted with 25 % acetonitrile in 10 mM H_3_PO_4_ at 5 mL/min. Retention times were 9.2 min for [^18^F]FPr-AMC15 and 11.0 min for [^18^F]FBu-AMC15.

Non-carrier-added [^18^F]FEt-AMC15 was found to decompose on the silica plate, so before TLC runs with [^18^F]FEt-AMC15 and its longer-chain homologues (including the analysis of plasma and tissue extracts), the start positions of the lanes on the TLC plate were pre-treated with AMC15 oxalate standard aqueous solution (2 μg in 2 μL) to hamper the on-plate decomposition.

#### Characterization and identity confirmation of tracers

All tracers were characterized by analytical radio-HPLC and radio-TLC. Conditions and results of the characterization are presented in Additional file 1: Table S1.

The presence of the corresponding “carrier” (i.e., tracer molecules that contain a [^19^F]-atom) in the formulated tracer solutions was confirmed by high performance liquid chromatography-electron spray tandem mass spectrometry (LC-ESI-MS/MS) analysis with the monitoring of fragmentation reactions of the tracer molecules. For all radioligands, ion current peaks with expected M/z ratios giving expected fragmentation products were observed (Additional file 1: Table S2). The identity of [^18^F]FEt-AMC13, [^18^F]FBu-AMC15, and [^18^F]FEt-AMC15 was also confirmed by co-elution with reference products.

#### LogD measurement

LogD of the tracers was measured using the radioactivity remaining on the HLB SPE cartridges after their elution with ethanol during the tracer formulation procedure (see above). The cartridge was dried with nitrogen for 5 min and eluted with at least 1.5 mL *n*-octanol. A 500 μL portion of octanol eluate was mixed with 500 μL of 1 M phosphate buffer (pH 7.4) and vigorously vortexed for 5 min. The mixture was then centrifuged at 17,000*g* for 10 min and 3 × 100 μL aliquots of organic and aqueous phase were loaded into a gamma-counter for radioactivity measurement. To correct for possible presence of radioactive impurities in the tracer, aliquots of organic and aqueous phases were later analyzed by radio-TLC, and the counts obtained from the gamma-counter were corrected according to the percentage of impurities found in aqueous and organic phases. LogD was calculated with the following formula:

$$ \mathrm{LogD}= \lg \left(\frac{{\mathrm{CPM}}_{\mathrm{org}} \times {X}_{\mathrm{org}}^{\mathrm{tracer}}}{{\mathrm{CPM}}_{\mathrm{aq}} \times {X}_{\mathrm{aq}}^{\mathrm{tracer}}}\right), $$

where CPM_org_ is the mean radioactivity of the organic aliquots, ${X}_{\mathrm{org}}^{\mathrm{tracer}}$ is the mean percentage of radioactivity accounting for intact tracer in organic aliquots (as determined from radio-TLC), and CPM_aq_ and ${X}_{\mathrm{aq}}^{\mathrm{tracer}}$ are the corresponding values for the aqueous phase.

For each tracer batch used for logD determination, the experiment was performed in duplicate. LogD values are means calculated from at least two independent experiments.

### In vitro autoradiography

In vitro autoradiography assay was performed as previously described [24]. Frozen brains of young (10–12 weeks of age; 300–350 g body weight) male Sprague-Dawley rats (Harlan, Netherlands) were cut at −12 °C into sagittal slices 20 μm thick using a Leica microtome, and the slices were thaw-mounted on Superfrost (70 × 22 mm, Fischer) adhesive slides. Only slices containing both striatal and cerebellar regions were used. The slides with slices mounted them were allowed to dry, then put into storage boxes with silica gel bags and stored at −80 °C.

On the day of the experiment (within 1 week from the preparation of the slices), the slides were taken out of storage and allowed to come to room temperature for 5–10 min. Then, 1–1.2 mL of incubation buffer (50 mM Tris-HCl, 5 mM KCl, 2 mM CaCl_2_, 2 mM MgCl_2_, 120 mM NaCl, pH 7.4 25 °C) was applied per slide, and the slides were pre-incubated for 15 min at room temperature. The buffer was then removed, and the slides were placed into staining jars containing radioligand solution (concentration range of 0.1–10 nM) in the incubation buffer with or without 10 μM raclopride (D_2/3_ antagonist) or 100 μM guanosine-5'-triphosphate sodium salt (GTP, stimulator of G-protein uncoupling from the receptors). After 35 min of incubation at 37 °C, slides were washed once with ice-cold incubation buffer (3.5 min) and dipped for 30 s into ice-cold distilled water to remove buffer salts.

After drying the slides in a stream of room-temperature air, they were exposed on phosphor storage screens for 6–10 h. The storage screens were read by Cyclone Storage Phosphor System (Packard Instruments Co.). Quantification of plate readings was done with OptiQuant software (version 3.00, Packard Instruments Co.).

### MicroPET study in rats

Three radioligands were evaluated in vivo: the originally developed [^18^F]FBu-AMC13 and [^18^F]FEt-AMC15, and [^18^F]FEt-AMC13, the homologue that showed the best results in the in vitro autoradiography experiments (see “Results” section).

Animal experiments were performed by licensed investigators in compliance with the Law on Animal Experiments of The Netherlands. The protocol was approved by the Committee on Animal Ethics of the University of Groningen. Young male (10–12 weeks of age, 300–350 g body weight) Sprague-Dawley rats (Harlan, the Netherlands) were used for all experiments. The rats were maintained at a 12-h light/12-h dark regime and were fed standard laboratory chow ad libitum.

Distribution of the radiotracers was studied in rats pre-treated, at random, with physiological saline (controls) or raclopride tartrate (1 mg/kg calculated per raclopride base, equivalent to 2.9 μmol/kg). Saline and raclopride were administered intravenously (1 mL/kg injected volume).

An additional group of saline pre-treated rats, injected with [^11^C]raclopride, served as positive controls for D_2/3_R-specific brain uptake.

Two rats were scanned simultaneously. Before all manipulations, animals were anesthetized with a mixture of isoflurane/air (inhalation anesthesia, 5 % ratio during induction, 2 % at maintenance). Cannulae were placed into the rats’ left femoral arteries and veins; the operation took 45–50 min. The rats were positioned supine inside the camera (Focus 220 microPET, Siemens-Concorde), one above the other with their heads in the camera’s field of view. A 515-s transmission scan with a Co-57 point source was performed. Tracer was injected through the venous cannula as a slow bolus (1 mL volume, 60 s long) using an infusion pump. The second (upper) animal was injected 16 min after the first (lower). Injected activities and doses are given in Table 1.

**Table 1:** Injection data for the PET study

Tracer	Injected activity, MBq/rat	Injected dose, nmol/kg	Rats per treatment group (saline/raclopride)	Pre-treatment time interval, min
[^18^F]FEt-AMC13	9.4 ± 1.2	0.55 ± 0.14	4/4	47 ± 27
[^18^F]FBu-AMC13	20.8 ± 10.6	0.45 ± 0.26, 10.4, 11.0^a^	3/3	52 ± 19
[^18^F]FEt-AMC15	26.6 ± 15.2	1.06 ± 0.65	3/3	48 ± 10
[^11^C]raclopride	16.9 ± 5.3	1.26 ± 0.16	4 (saline only)	36 ± 3

One rat in the saline-treated group and one rat in the raclopride-treated group were injected, respectively, with 10.4 and 11.0 nmol/kg tracer. As all outcome measures (time-activity curves, uptake ratios) obtained from these animals were very close to the results from other animals in the corresponding groups, these results were not omitted or separated, but combined with the rest of the data

PET acquisition was started during the injection of radioactivity in the first rat. A 106-min long (76 min long in the case of [^11^C]raclopride) list-mode acquisition protocol was used for the scan.

Throughout the scan, arterial blood samples (15–19 samples; 0.10–0.15 mL each) were withdrawn from each rat through the arterial cannula. Time intervals between samples gradually increased from 5–10 s right after injection to 30 min at the end of the scan. From each sample, 25 μL aliquots of whole blood were withdrawn. The rest was centrifuged at 3500 g for 5 min; 25 μL supernatant (plasma) aliquots were withdrawn and deproteinated with 75 μL of ice-cold acetonitrile. From the resulting 100 μL, 2–8 μL portions (i.e., 2–8 % of volume) were taken for thin layer chromatography analysis (radio-TLC) to assess radiometabolite content.

The radioactivity of the plasma and whole blood samples was measured using a well-type gamma-counter (LKB-1282-Compugamma, LKB Wallac).

Radio-TLC was performed on silica plates. Samples of the formulated tracers (diluted) were run along with deproteinated plasma samples to confirm the identity of the parent compound in plasma. Eluent systems used and TLC R_f_ values recorded are presented in Additional file 1: Table S1.

After the end of the PET scans (i.e., 98 ± 8 min after tracer injection), anesthetized animals were sacrificed by heart extirpation and dissected. Samples of brain and peripheral tissues were taken. All samples were weighed, and their radioactivity was measured in a gamma-counter.

### MicroPET image reconstruction and data analysis

List-mode data from the 106-min-long and 76-min-long scans were reframed into, respectively, 90-min-long and 60-min-long dynamic sequences of 6 × 10, 4 × 30, 2 × 60, 1 × 120, 1 × 180, 4 × 300, 3 × 600, and (for 90-min-long scans) 2 × 900 s frames. The data were reconstructed per time frame using an iterative reconstruction algorithm (attenuation-weighted 2-dimensional ordered-subset expectation maximization, provided by Siemens; 4 iterations, 16 subsets; zoom factor 2). Datasets were fully corrected for random coincidences, scatter, and attenuation. Data from the transmission scan were used for attenuation correction. The final datasets consisted of 95 slices, with a slice thickness of 0.8 mm and an in-plane image matrix size of 128 × 128 and pixel size of 0.47 × 0.47 mm.

Reconstructed images were analyzed with Inveon 2.0 software (Siemens Medical Solutions, USA, Inc). Regions of interest (ROIs) were drawn manually on a T_2_-weighted MRI template of rat brain around the striatum, brainstem, cortex, hippocampus, hypothalamus, thalamus, olfactory bulbs, and cerebellum around the whole brain and around the pituitary gland. The MRI template was co-registered with the PET scan by image fusion. The time-activity curves (TAC) per ROI were determined in Bq/cm^3^ units and converted into standardized uptake values (SUVs).

Kinetic analysis is described using the nomenclature proposed by Innis et al. [26]. ROI TACs of [^18^F]FEt-AMC13 were analyzed with one-tissue and two-tissue compartmental models of reversible binding (1TCM and 2TCM, respectively) to obtain individual rate constants. From these, the regional distribution volumes (*V*_T_) were calculated and compared to those obtained with Logan graphical analysis. Binding potentials (BP_ND_) were calculated from the distribution ratios as *V*_T_ (target) / *V*_T_ (cerebellum) – 1, or estimated with the simplified reference tissue model (SRTM) using the cerebellum as reference region.

Metabolite-corrected plasma-derived arterial input function and whole blood time-activity curve were used for Logan, 1TCM, and 2TCM analyses. Fractional cerebral blood volume was set to 3.6 % [27]. For one rat in the raclopride-treated group, no input curves could be obtained; therefore, its PET data were only analyzed with SRTM.

### Ex vivo autoradiography study in rats

To further assess the specific binding of [^18^F]FEt-AMC13 to the striatal D_2/3_R, two groups of rats were anesthetized, pre-treated with saline (*n* = 3) or raclopride (*n* = 3), and 26 ± 3 min later injected with a bolus of [^18^F]FEt-AMC13 (4.2 ± 1.4 MBq, 0.14 ± 0.04 nmol/kg) into the penile vein. Rats were sacrificed by heart extirpation 35 min later. Brains were quickly extracted and separated into two halves along the sagittal symmetry plane or along the coronal plane spanning the thalamus.

One half of the brain (rostral or randomly taken left/right) was quickly frozen over liquid nitrogen and prepared for cutting on the microtome in the same manner as brains used for in vitro autoradiography [24]. Slices 40 μm thick were cut, mounted on Superfrost glass slides, permitted to dry, and then directly applied to the phosphor storage screens.

Another half of the brain was homogenized in 3 mL of ice-cold acetonitrile using Heidolph IDAX600 homogenizer at maximum speed. Homogenate was centrifuged at 3500*g* for 5 min, and the supernatant (containing >95 % total radioactivity) was analyzed by radio-TLC in the same way as described above for the plasma.

### D_2/3_R-specific binding quantification

Degree of D_2/3_R-specific binding of the tracers was characterized by BP_ND_ (as given above) or the specific binding ratio (SBR) given as target region activity/cerebellar activity - 1. Both were calculated under control conditions and in response to the raclopride challenge.

### Statistics

All data are presented as means ± standard deviations. Comparison of means was done using 2-sided unpaired Welch *t* test. *p* values below 0.05 were considered significant. No multiple comparison corrections were performed.

## Results

### Preparation of radioligands

The labeling scheme used for the preparation of the tracers is given in Fig. 2. Decay-corrected yields, radiochemical purities, specific activities, and logD values of the prepared tracers are listed in Table 2. Radio-HPLC and radio-TLC characterization data of the prepared tracers can be found in Additional file 1: Table S1. LC-ESI-MS/MS characterization data of the [^19^F]-carrier compounds in the decayed samples of purified tracers are described in Additional file 1: Table S2.

**Table 2:** Synthetic and analytical data for [^18^F]AMC13 and [^18^F]AMC15 homologues

Compound	Isolated radiochemical yield, %	Radiochemical purity, %	End-of-synthesis specific activity, GBq/μmol	logD
[^18^F]FEt-AMC13	12 ± 6	>95	102 ± 30	1.67 ± 0.07
[^18^F]FPr-AMC13	7 ± 6	>95	29 ± 27	1.98 ± 0.08
[^18^F]FBu-AMC13	8 ± 7	93 ± 2	85 ± 83	2.50 ± 0.06
[^18^F]FEt-AMC15	5 ± 3	93 ± 2	25 ± 15	1.48 ± 0.002
[^18^F]FPr-AMC15	3 ± 1	93 ± 5	9 ± 7	2.39 ± 0.001
[^18^F]FBu-AMC15	6 ± 0.3	93 ± 6	10 ± 3	2.71 ± 0.07

Values are means ± SD of ≥3 determinations from ≥2 independent experiments

For both [^18^F]AMC13 and [^18^F]AMC15 homologues, reverse-phase radio-HPLC retention times, normal-phase TLC retention factors, and logD values increased with the length of the [^18^F]fluoroalkyl chain.

### In vitro autoradiography

**Fig. 3:**
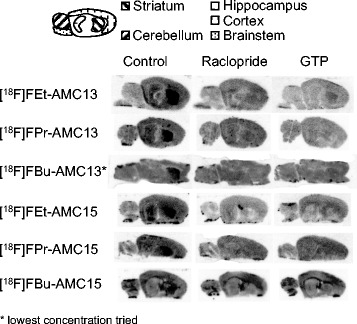
Representative in vitro autoradiography images of rat brain slices for the assayed radioligands. *Left column*—slides incubated with radioligand only. *Middle column*—radioligand and 10 μM raclopride. *Right column*—radioligand and 100 μM GTP

All [^18^F]fluoroethyl and [^18^F]fluoropropyl compounds showed preferential uptake in the D_2/3_R-rich striatum (Fig. 3). [^18^F]FEt-AMC13 had the highest striatal SBR, followed by [^18^F]FPr-AMC13 and [^18^F]FEt-AMC15 (Table 3). [^18^F]FBu-AMC13 only showed discernible specific binding at the lowest concentration tested.

**Table 3:** Quantitative results of in vitro autoradiography

Tracer	Tracer concentration, nM^a^	Striatal SBR^b^ (% change relative to control condition)		
		Control	Raclopride	GTP
[^18^F]FEt-AMC13	0.9 ± 0.7 (*n* = 3)	3.40 ± 1.25	0.63 ± 0.53 (−81%)*	0.66 ± 0.52 (−81%)*
[^18^F]FPr-AMC13	1.4 ± 1.6 (*n* = 3)	2.07 ± 1.06	0.41 ± 0.23 (−80%)*	0.42 ± 0.23 (−80%)*
[^18^F]FBu-AMC13^c^	0.2 (*n* = 1)	0.85 ± 0.23	0.10 ± 0.19 (−88%)*	0.20 ± 0.16 (−76%)*
	4.5 ± 3.6 (*n* = 3)	0.19 ± 0.18	0.04 ± 0.22 (−79%)	–
[^18^F]FEt-AMC15^c^	5.8 ± 1.6 (*n* = 4)	1.64 ± 1.04	0.65 ± 0.37 (−60%)*	0.67 ± 0.28 (−59%)*
[^18^F]FPr-AMC15	5.0 ± 7.2 (*n* = 3)	1.01 ± 0.46	0.39 ± 0.22 (−61%)*	0.37 ± 0.24 (−63%)*
[^18^F]FBu-AMC15	2.3 ± 1.8 (*n* = 3)	0.69 ± 0.30	0.25 ± 0.23 (−64%)*	0.23 ± 0.23 (−67%)*

*p < 0.05 relative to control values, 2-sided Welch test

^a^Concentrations are means ± SD from *n* independent experiments

^b^Specific binding ratio (SBR) values are means ± SD from the total number of slides (3–28 per condition) assayed in all experiments

^c^Published earlier [24]

In the presence of 10 μM of raclopride, SBR values of all AMC13 homologues decreased by 79–88 %, and those of all AMC15 homologues by 60–64 % compared to control conditions. GTP decreased the SBR values to the same extent, implying that the striatal binding of all tracers was predominantly to the high-affinity subset of the D_2/3_Rs (Table 3).

### In vivo and ex vivo brain uptake of the tracers under control conditions

**Fig. 4:**
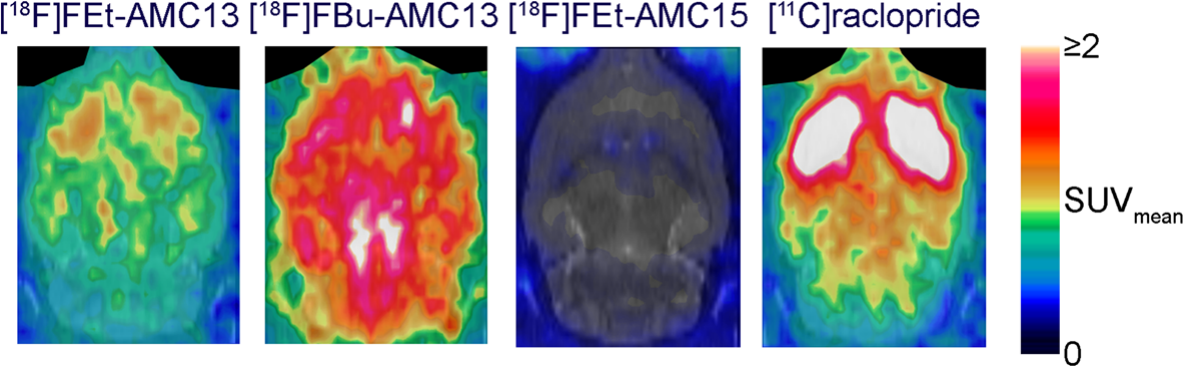
Representative PET images for three [^18^F]fluoroalkyl-AMC radioligands and [^11^C]raclopride acquired in rats. Images are summed from 5 min post-injection till the end of scan. Harderian glands (*on top*) are masked

[^18^F]FEt-AMC13 and [^18^F]FBu-AMC13 showed good blood-brain barrier (BBB) penetration (Fig. 4). Brain uptake peaked at 0.7 % injected dose (ID) and 0.9 % ID, respectively, 3 min after radiotracer injection.

**Fig. 5:**
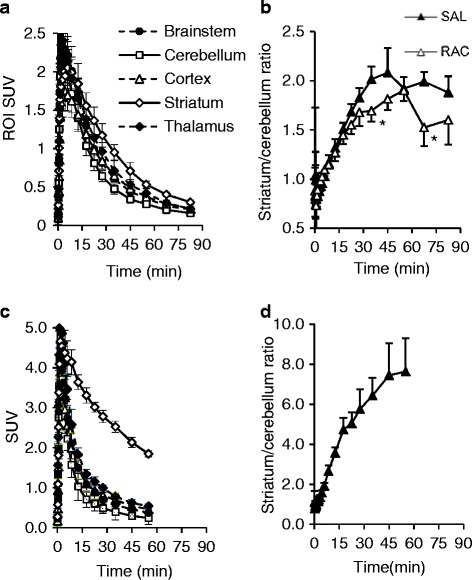
Uptake of [^18^F]FEt-AMC13 (**a**, **b**) and [^11^C]raclopride (**c**, **d**) in rat brain. **a**, **c** Region-of-interest time-activity curves of control rats. **b**, **d** Striatum-to-cerebellum ratios in rats pre-treated with saline (SAL) and 1 mg/kg (2.9 μmol/kg) raclopride (RAC). Points represent group means, *error bars* show standard deviations

For both tracers, the brain region with the lowest uptake was the cerebellum. [^18^F]FBu-AMC13 had the highest uptake in the brainstem, followed by the striatum; striatum-to-cerebellum uptake ratio peaked at 1.41 ± 0.08 (Additional file 1: Figure S1. [^18^F]FEt-AMC13 and [^11^C]raclopride had the highest uptake in the striatum (Fig. 5). Striatum-to-cerebellum uptake ratio of [^18^F]FEt-AMC13 peaked 45 min post-injection at 2.08 ± 0.25 (Fig. 5b); the same ratio measured by ex vivo autoradiography was 2.81 ± 0.29 (35 min post-injection). The striatum-to-cerebellum ratio of [^11^C]raclopride was 7.45 ± 1.60 (45 min post-injection; Fig. 5d).

[^18^F]FEt-AMC15 essentially did not penetrate the BBB. Its in vivo brain uptake peaked at 0.2 % ID 3 min post-injection and fell to 0.04 % ID 60 min post-injection.

Ex vivo measurements of the uptake of the tracers in the brain and peripheral tissues performed after the PET scan are given, respectively, in Table 4 and Additional file 1: Table S3.

**Table 4:** Ex vivo uptake of the evaluated tracers in brain regions and skull bones of rats

Tracer	[^18^F]FEt-AMC13		[^18^F]FBu-AMC13		[^18^F]FEt-AMC15	
Treatment	Saline	Raclopride	Saline	Raclopride	Saline	Raclopride
Region	(*n* = 4)	(*n* = 4)	(*n* = 3)	(*n* = 3)	(*n* = 3)	(*n* = 3)
Striatum	0.28 ± 0.03 (1)	0.23 ± 0.08	1.60 ± 0.33 (5)	1.21 ± 0.14	0.02 ± 0.02	0.03 ± 0.01
Hippocampus	0.22 ± 0.02 (2)	0.23 ± 0.08	1.50 ± 0.25 (6)	1.21 ± 0.06	0.03 ± 0.02	0.03 ± 0.01
Thalamus + hypothalamus	0.19 ± 0.003 (4)	0.19 ± 0.05	2.07 ± 0.70 (1)	1.07 ± 0.10	0.03 ± 0.01	0.03 ± 0.01
Cortex	0.18 ± 0.01 (5)	0.16 ± 0.04	1.50 ± 0.32 (7)	1.01 ± 0.11	0.03 ± 0.003	0.03 ± 0.01
Brainstem	0.18 ± 0.02 (6)	0.16 ± 0.04	1.97 ± 0.42 (2)	1.25 ± 0.14	0.03 ± 0.004	0.03 ± 0.01
Olfactory bulbs	0.17 ± 0.01 (7)	0.15 ± 0.04	1.85 ± 0.64 (3)	0.86 ± 0.09	0.03 ± 0.001	0.03 ± 0.02
Rest of brain	0.20 ± 0.02 (3)	0.18 ± 0.03	1.60 ± 0.25 (4)	1.10 ± 0.17	0.02 ± 0.02	0.03 ± 0.02
Cerebellum	0.13 ± 0.01 (8)	0.13 ± 0.02	1.11 ± 0.29 (8)	0.73 ± 0.08	0.03 ± 0.01	0.03 ± 0.01
Mean brain uptake	0.18 ± 0.01	0.16 ± 0.04	1.56 ± 0.33	1.03 ± 0.11	0.03 ± 0.01	0.03 ± 0.01
Pituitary	0.76 ± 0.08	0.59 ± 0.20	3.19 ± 0.29	2.72 ± 0.77	1.36 ± 0.30	1.08 ± 0.27
Parietal bone	0.10 ± 0.01	0.10 ± 0.01	0.61 ± 0.17	0.70 ± 0.18	0.09 ± 0.03	0.08 ± 0.03

Data are presented as means ± standard deviations of standard uptake values. The ranking of the uptake magnitude (highest to lowest) among brain regions within the BBB is shown in parentheses (only for [^18^F]AMC13 homologues)

### Tracer metabolism

**Fig. 6:**
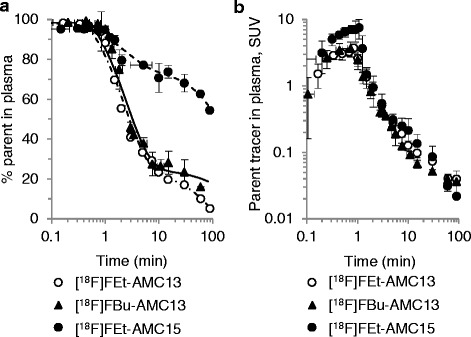
Metabolism and pharmacokinetics of [^18^F]FEt-AMC13, [^18^F]FBu-AMC13, and [^18^F]FEt-AMC15 in the plasma of living rats. **a** Percentage of intact tracer in the total plasma radioactivity. **b** Concentration of intact tracer in the plasma (i.e., metabolite-corrected plasma input curves). Points represent group means, *error bars* show standard deviations. Horizontal axes show time after tracer injection

[^18^F]FEt-AMC13 and [^18^F]FBu-AMC13 were quickly metabolized in the plasma, while [^18^F]FEt-AMC15 was more stable (Fig. 6). Tracers were eliminated from the plasma with half-lives of 3 to 12 min. Radiometabolites of all tracers in the plasma and in the brain tissue extract were considerably more hydrophilic than their corresponding parent compounds, as they all had TLC R_f_ values of 0, compared to R_f_ of 0.16–0.56 for the tracers themselves (Additional file 1: Table S1). Intact [^18^F]FEt-AMC13 constituted 92 % of radioactivity from brain tissue extract 35 min post-injection (Additional file 1: Figure S2).

Uptake in the parietal bone was lower than in the brain tissue for all [^18^F]AMC13 homologues (Table 4), suggesting low levels of in vivo defluorination.

### Kinetic modeling for [^18^F]FEt-AMC13

2TCM and SRTM fits of ROI TACs were consistently better than 1TCM fits (Additional file 1: Figure S3) as assessed by Akaike’s information criterion, but the estimates of individual rate constants of 2TCM were highly variable and often physiologically implausible (Additional file 1: Table S4).

The most robust ROI *V*_T_ estimates were obtained from the Logan analysis (Additional file 1: Table S5). Logan *V*_T_ values correlated better with 2TCM *V*_T_ (*r* = 0.97) than with 1TCM *V*_T_ values (*r* = 0.89; Additional file 1: Figure S4). No robust BP_ND_ estimates could be obtained from the k_3_/k_4_ rate constant ratio of 2TCM (data not shown), but BP_ND_ estimates calculated from 1TCM, 2TCM, and Logan *V*_T_ data and obtained by SRTM correlated well with each other for all regions except the pituitary and olfactory bulbs (Additional file 1: Figure S5).

The highest BP_ND_ values in control rats were found in the striatum (1TCM 0.63 ± 0.08; 2TCM 0.56 ± 0.05, Logan 0.52 ± 0.01; SRTM 0.51 ± 0.02).

### Blocking experiments

Pre-treatment with 1 mg/kg (2.9 μmol/kg) raclopride decreased peak PET-based striatal SBR of [^18^F]FEt-AMC13 by 17 % (from 1.08 ± 0.25 to 0.90 ± 0.11), though the SBR change was not statistically significant (*p* = 0.25). Striatal SBR based on after-scan biodistribution measurements decreased by 31 % (from 1.07 ± 0.10 to 0.74 ± 0.27), but was also not statistically significant (*p* = 0.17). Ex vivo autoradiography-based SBR fell by 39 % (from 1.81 ± 0.29 to 1.06 ± 0.17), which was statistically significant (*p* < 0.01), and the blocking of D_2/3_R specific striatal binding was clearly visible on the autoradiography images (Additional file 1: Figure S6).

Striatal BP_ND_ fell by 18–27 % (*p* < 0.05 for 2TCM, Logan and SRTM; see Table 5 and Additional file 1: Table S6) depending on the kinetic model used. Modified Lassen plot analysis [28] estimated the raclopride-induced D_2/3_R occupancy at 20–25 % (*p* < 0.05 for 1TCM and 2TCM), while 41–55 % of striatal *V*_T_ and 11–26 % of cerebellar *V*_T_ were estimated to be potentially displaceable, i.e., represent D_2/3_R specific binding (Additional file 1: Figure S7).

**Table 5:** 2TCM and SRTM-derived BP_ND_ of [^18^F]FEt-AMC13 per brain region in control and raclopride-treated rats

Region of interest	2TCM BP_ND_ (*V*_T_-based)		SRTM BP_ND_	
	Control	Raclopride	Control	Raclopride
Striatum	0.56 ± 0.05	0.41 ± 0.05 (−27%)*	0.51 ± 0.02	0.38 ± 0.05 (−26%)**
Hippocampus	0.30 ± 0.07	0.26 ± 0.06 (−13%)	0.26 ± 0.04	0.23 ± 0.05 (−13%)
Thalamus	0.33 ± 0.02	0.30 ± 0.01 (−10%)*	0.28 ± 0.06	0.26 ± 0.02 (−7%)
Hypothalamus	0.22 ± 0.03	0.18 ± 0.06 (−19%)	0.21 ± 0.03	0.16 ± 0.04 (−23%)
Cortex	0.13 ± 0.04	0.10 ± 0.02 (−27%)	0.11 ± 0.03	0.08 ± 0.02 (−24%)
Brainstem	0.22 ± 0.02	0.10 ± 0.02 (−53%)**	0.18 ± 0.04	0.07 ± 0.03 (−60%)**
Olfactory bulbs	0.08 ± 0.13	0.01 ± 0.04 (−88%)	0.10 ± 0.13	0.11 ± 0.05 (+10%)
Pituitary	0.36 ± 0.07	0.13 ± 0.04 (−23%)	0.71 ± 0.32	0.55 ± 0.53 (−22%)

Data are presented as means ± SD, *n* = 4 for the control group and *n* = 3 for the raclopride-treated group (except for the SRTM BP_ND_ values, where *n* = 4). Next to the BP_ND_ values of the raclopride-treated group, percentage of change relative to the control group is shown

*p < 0.05; **p < 0.01, 2-sided Welch test

For [^18^F]FBu-AMC13 pre-treatment with raclopride, decreased absolute uptake in all brain regions, and striatum-to-cerebellum uptake ratios were higher in raclopride-treated group than in the saline-treated group (Additional file 1: Figure S1c).

For [^18^F]FEt-AMC15, pre-treatment with raclopride caused no detectable alterations of radioactivity distribution in the brain (data not shown).

## Discussion

We have previously described the design, synthesis, and in vitro evaluation of a series of high-affinity D_2/3_ agonists based on the AMC scaffold. Two compounds from that series, FBu-AMC13 and FEt-AMC15, were confirmed to have high-affinity towards D_2/3_R and act as agonists at D_2_R [24]. They were [^18^F]-labeled and showed favorable results in the in vitro autoradiography experiments. In this study, we have evaluated these compounds in vivo and further optimized their characteristics by shortening or lengthening the [^18^F]fluoroalkyl chains in their structures. We did not directly measure the affinities and intrinsic activities of the shorter-chain homologues of FBu-AMC13 and longer-chain homologues of FEt-AMC15 towards D_2/3_R, assuming that changing the fluoroalkyl chain would have negligible influence on these parameters, because this fluoroalkyl chain is situated outside the main AMC pharmacophore [24].

Both raclopride and GTP decreased striatal SBRs of all tracers significantly and to a similar extent, which was consistent across the homologous series (Table 3). This indicates that all our tracers specifically bind to striatal dopamine D_2/3_ receptors in their G-protein-coupled “high-affinity state.” Tracers with a shorter alkyl chain showed higher in vitro autoradiography-based striatal SBR values (Et > Pr > Bu). These findings are in agreement with our hypothesis that varying the [^18^F]fluoroalkyl chain length would influence non-specific binding, but not the D_2/3_R-affinity or agonism of the tracers.

In vivo, [^18^F]FEt-AMC15 showed very low levels of BBB penetration, which may be the consequence of the tracer’s low lipophilicity and/or may imply that it is a substrate for one of the drug efflux pump proteins such as P-glycoprotein. Meanwhile, [^18^F]FEt-AMC13, which is the short-alkyl-chain homologue of [^18^F]FBu-AMC13, showed greatly improved specific-to-non-specific binding ratios compared to the “original” radioligand, while retaining good brain penetration and favorable metabolic profile.

Our approach has proven to be a useful and relatively simple tactic for the adjustment of the radioligands’ lipophilicity with the goal of optimizing their non-specific binding and brain penetration.

The time-activity curves of [^18^F]FEt-AMC13 were better approximated with the 2TCM model than with 1TCM model, and 2TCM-derived *V*_T_ and BP_ND_ estimates correlated better than 1TCM-derived *V*_T_ and BP_ND_ estimates with the same data obtained from Logan analysis that makes no assumptions regarding the number of kinetic compartments. However, the 2TCM model did not always provide reliable estimates of the individual rate constants of [^18^F]FEt-AMC13 uptake in the brain. This may have been caused by the quality of input data: insufficient counts or imprecise estimates of the plasma input can make the optimization routine converge at a local minimum. Fortunately, the estimates of *V*_T_ and BP_ND_ were more robust and consistent, so these were used for the evaluation of [^18^F]FEt-AMC13.

Estimation of the magnitude of the D_2/3_R blockade induced by 1 mg/kg (2.9 μmol/kg) raclopride in rats’ striata in vivo performed with the use of different approaches produced consistent results: changes in striatal BP_ND_ and (PET and autoradiography-derived) SBR were similar to the modified Lassen plot-based estimate of D_2/3_R occupancy by raclopride. Depending on the estimate, raclopride pre-treatment blocked 17–39 % of D_2/3_R-specific signal in the striatum. Though a bolus dose injected in an in vivo experiment cannot be directly compared with ligand concentration applied in an in vitro experiment, our in vivo findings are also arguably consistent with our in vitro findings, where a higher concentration of raclopride (10 μmol/L) used in the in vitro autoradiography study induced a greater decrease (81 %) in striatal SBR in rat brain slices compared to control conditions.

Our in vivo findings, however, do not agree with the published studies which reported >90 % displacement of apparent D_2/3_R-specific striatal uptake of [^11^C]MNPA and [^11^C]PHNO in Sprague-Dawley rats with 1–2 mg/kg (2.9–5.8 μmol/kg) raclopride [29–31]. Even a fivefold lower dose (0.2 mg/kg, 0.58 μmol/kg) displaced over 80 % of apparent D_2/3_R-specific [^11^C](+)PHNO uptake [30]. It is not clear why the apparent displacement of [^18^F]FEt-AMC13 from the striatum by 1 mg/kg raclopride is lower than what is reported for other D_2/3_R tracers in the literature. It is unlikely that we violated the so-called tracer conditions because crude estimation of the D_2/3_R occupancy by [^18^F]FEt-AMC13 itself using the approach described by Skinbjerg et al. [32] produced the values of no more than 3 % in all our experiments. It may be that the signal-to-noise ratio in our data was insufficient, which made it difficult to precisely estimate the contribution of specific binding to total tracer binding in various ROIs. The presence of specific binding of [^18^F]FEt-AMC13 to sites other than D_2/3_R, which has a comparatively high density in the striatum, may also be implied. We have previously demonstrated that FBu-AMC13, a longer-chain homologue of FEt-AMC13, has low affinity towards D_1_ receptors (7 μM on average) [24], while (*R*)-2-[(benzylamino)methyl]chroman-7-ol and 2-[(4-hydroxybenzylamino)methyl]chroman-7-ol, compounds structurally closely related to FEt-AMC13, were shown by Mewshaw and co-workers to be selective for D_2/3_R against serotonin-1A and adrenergic α_1_ receptors [23]. In the in vitro autoradiography study, presence of 10 μmol/L raclopride decreased striatal SBR of [^18^F]FEt-AMC13 by 81 %, which also does not support the hypothesis of the existence of significant non-D_2/3_R-specific binding of [^18^F]FEt-AMC13 in the striatum. Still, a separate study of pharmacological selectivity of FEt-AMC13, including direct determination of the affinities of FEt-AMC13 towards high- and low-affinity states of D_2/3_R, is necessary to clarify this issue.

Apart from the striatum, the olfactory bulbs and pituitary gland are known to contain high densities of D_2/3_R available to agonist radioligands [33]. However, no blocking effect of raclopride could be observed in these regions (Tables 4 and 5), and the relatively high tracer uptake in the pituitary is more likely to be explained by the pituitary being situated outside the BBB than by the presence of D_2/3_R-specific binding of [^18^F]FEt-AMC13 there.

A striatum-to-cerebellum ratio of 2.08 is on par with the value of 2 reported in rats for [^18^F]5-OH-FPPAT [20] and with the value of 1.97 obtained for our other separately evaluated AMC derivative [^18^F]AMC20 [34]. However, the striatal BP_ND_ values of 0.51–0.63 obtained for [^18^F]FEt-AMC13 are lower than the values of at least 0.8–1.0 reported in rodents for existing [^11^C]-labeled D_2/3_ agonists [19, 29, 31]. Non-specific binding of the tracer in the tissue can slow down the association rate of the tracer-receptor binding by leaving less free tracer in the tissue. The launch of the canonical signaling cascade induced by agonist binding eventually leads to the uncoupling of the G-protein from the receptor and the relaxation of the receptor into its low-affinity state [18, 35], which may increase the effective dissociation rate of an agonist tracer from the receptors. To find out which factors limit the signal-to-noise ratio of [^18^F]FEt-AMC13, a more thorough study of its binding kinetics in a more controlled environment (e.g., in vitro) is necessary.

## Conclusions

We have evaluated two homologous series of agonist radiopharmaceuticals based on the AMC scaffold that bind with high affinity and selectivity to D_2/3_R in vitro. Varying the length of the [^18^F]fluoroalkyl group allowed us to significantly improve the specific-to-non-specific binding ratio of one of our original structures. The resulting ligand, [^18^F]FEt-AMC13, demonstrated specific binding to the striatal D_2/3_R in vitro as well as in vivo. In vitro findings also confirmed the tracer’s binding to the high-affinity state of D_2/3_R. Further investigation of the binding characteristics of FEt-AMC13 and optimization of its molecular structure can lead to an improved [^18^F]-labeled radioligand suitable for clinical D_2/3_R imaging by PET.

## Additional file

Additional file 1: **Supplementary material.** Supplemental tables and supplemental figures referred to in this article.
