# Supplementary material for: Synthesis and evaluation in rats of homologous series of [18F]-labeled dopamine D2/3 receptor agonists based on the 2-aminomethylchroman scaffold as potential PET tracers
Source: EJNMMI Res. 2015 Jul 25;5:41. doi: 10.1186/s13550-015-0119-x (PMC4512978; doi:10.1186/s13550-015-0119-x)
Supplement: Supplementary file 1 — Supplementary material. Supplemental tables and supplemental figures referred to in this article. [file 13550_2015_119_MOESM1_ESM.pdf]

SYNTHESIS AND EVALUATION IN RATS OF HOMOLOGOUS SERIES OF [^{18}F]-LABELED DOPAMINE $D_{2/3}$ RECEPTOR AGONISTS BASED ON THE 2-AMINOMETHYLCHROMAN SCAFFOLD AS POTENTIAL PET TRACERS

SUPPLEMENTARY MATERIAL

Vladimir Shalgunov[1], Jan-Peter van Wieringen[2], Henk M. Janssen[3], P. Michel Fransen[3], Rudi A.J.O. Dierckx[1], Martin C. Michel[4], Jan Booij[2], Philip H. Elsinga[1]*

[1]Department of Nuclear Medicine and Molecular Imaging, University Medical Center Groningen, University of Groningen, Groningen, The Netherlands

[2]Department of Nuclear Medicine, Academic Medical Center, University of Amsterdam, Amsterdam, The Netherlands

[3]SyMO-Chem BV, Eindhoven, The Netherlands

[4]Department of Pharmacology, Johannes Gutenberg University, Mainz, Germany

*Corresponding author:

Prof. Dr. Philip H. Elsinga

Department of Nuclear Medicine and Molecular Imaging

University Medical Center Groningen

University of Groningen

Hanzeplein 1

9713 GZ Groningen, the Netherlands

phone: +31-50-361-32-47

fax: +31-50-361-16-87

email: p.h.elsinga@umcg.nl

LIST OF ABBREVIATIONS

1TCM – one-tissue compartment model

2TCM – two-tissue compartment model

BP_{ND} – binding potential non-displaceable (equilibrium concentration ratio of specifically bound radioligand to non-displaceable radioligand in tissue)[1]

DMF – N,N'-dimethylformamide

ESI-MS – electron-spray ionization mass-spectrometry

GPCR – G-protein coupled receptor

GTP – guanosinetriphospate

HPLC – high-pressure liquid chromatography

LC – liquid chromatography

MS/MS – tandem mass spectrometry

PET – positron emission tomography

ROI – region of interest

SPECT – single photon emission computed tomography

SRTM – simplified reference-tissue model

SUV – standardized uptake value (regional uptake relative to mean uptake across the body)

TAC – time-activity curve

TLC – thin-layer chromatography

UV-VIS – ultraviolet/visible

V_T – total distribution volume of a radioligand in the tissue (i.e. the sum of distribution volumes of free, non-specifically and specifically bound radioligand)

SUPPLEMENTAL FIGURES

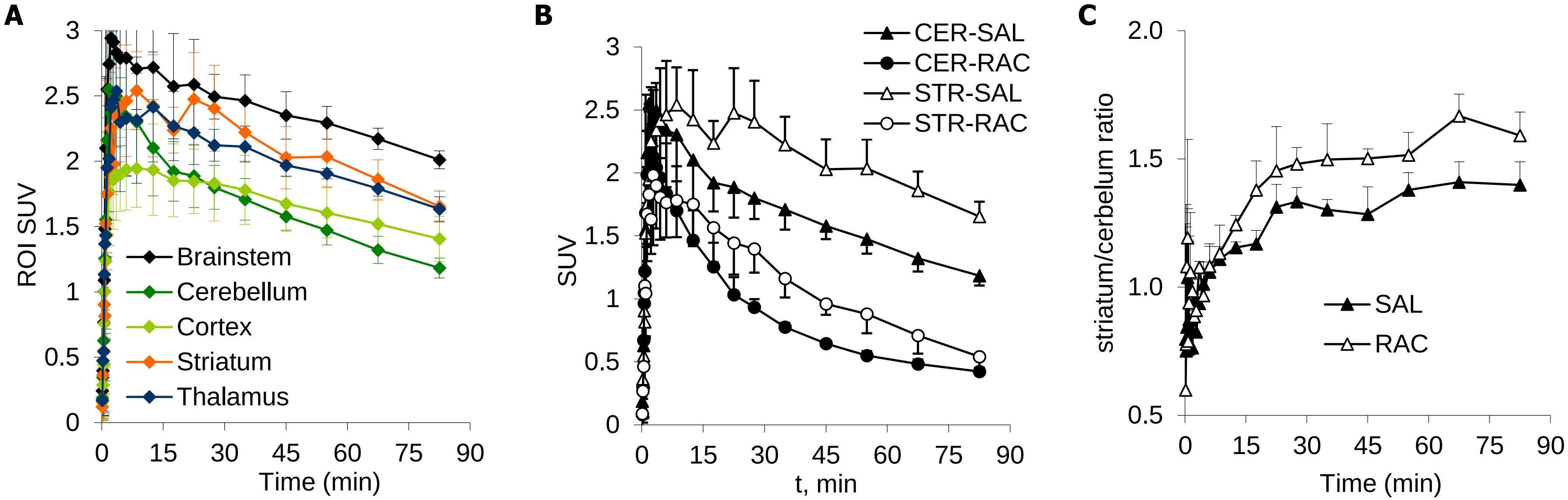

Supplemental Figure 1. Brain uptake (means±SD) of [^{18}F]FBu-AMC13 in rat brain. A – time-activity curves (TACs) per region-of-interest (ROI) in control (saline-treated) rats. B – cerebellar (CER) and striatal (STR) TACs of in saline-treated (SAL) and raclopride-treated (RAC) rats. C – striatum-to-cerebellum uptake ratios in saline-treated and raclopride-treated rats.

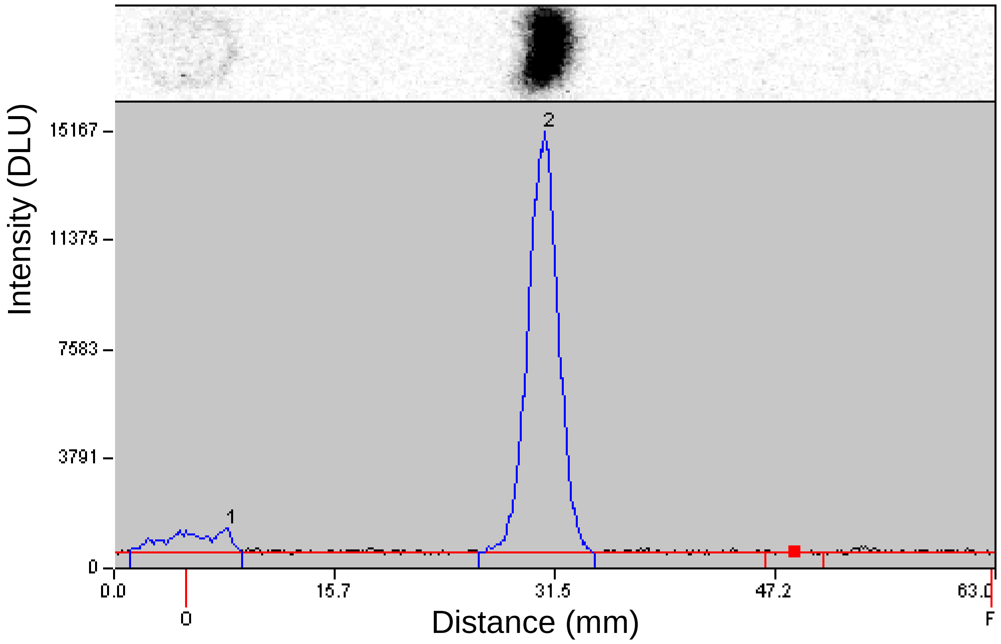

Supplemental Figure 2. Representative TLC of radioactivity extracted from the brain of a rat injected with [^{18}F]FEt-AMC13.

Peak 1 corresponds to radiometabolites remaining on the start. Peak 2 corresponds to intact [^{18}F]FEt-AMC13. Eluent is ethylacetate/methanol/triethylamine 100/5/1.

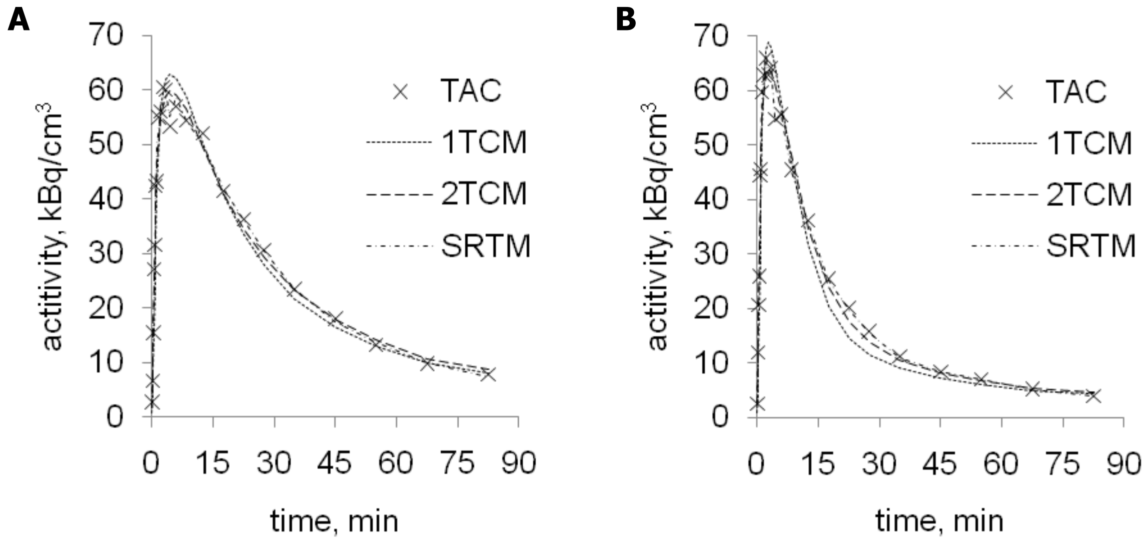

Supplemental Figure 3. Representative striatal (A) and cerebellar (B) TACs of [^{18}F]FEt-AMC13 for a control rat and the corresponding fits with 1TCM, 2TCM and SRTM models.

2TCM fits were better than 1TCM fits in 69 out of 70 (99%) cases, as assessed by Akaike's information criterion (AIC). SRTM fits had lower AIC value than 2TCM fits in 44 out of 70 (63%) cases.

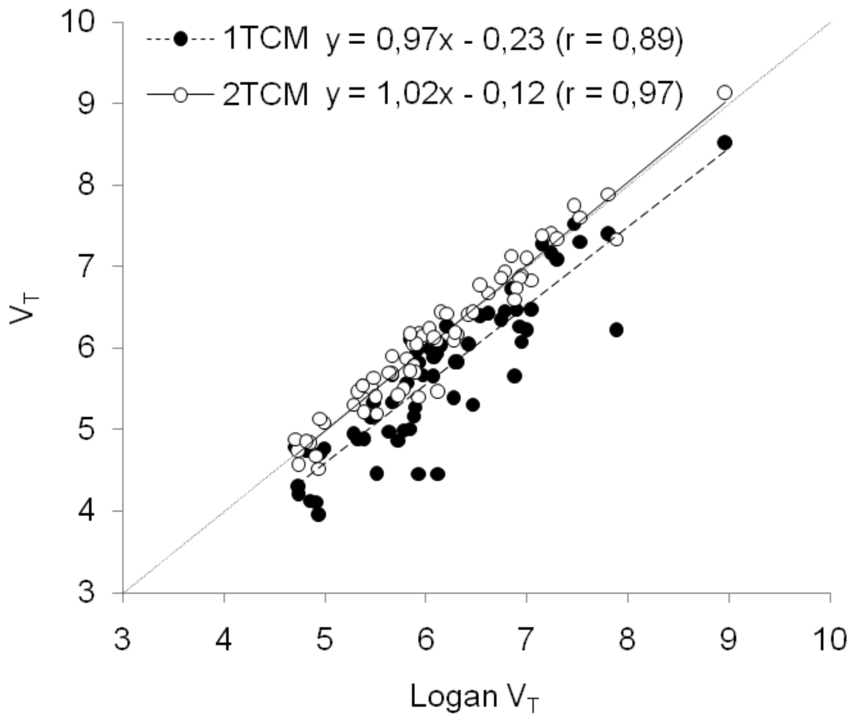

Supplemental Figure 4. Correlation of region-of-interest total distribution volumes (V_T) of [^{18}F]FEt-AMC13 obtained by Logan analysis with V_T values obtained by analyses with 1TCM and 2TCM models. Linear regression lines along with their equations and r and p values and the unity line (x=y) are shown on the graphs.

Points represent fits for individual regions in individual animals (both control and raclopride-pre-treated)**.** Data for striatum, hippocampus, thalamus, hypothalamus, cortex, brainstem, cerebellum, olfactory bulbs and pituitary are used.

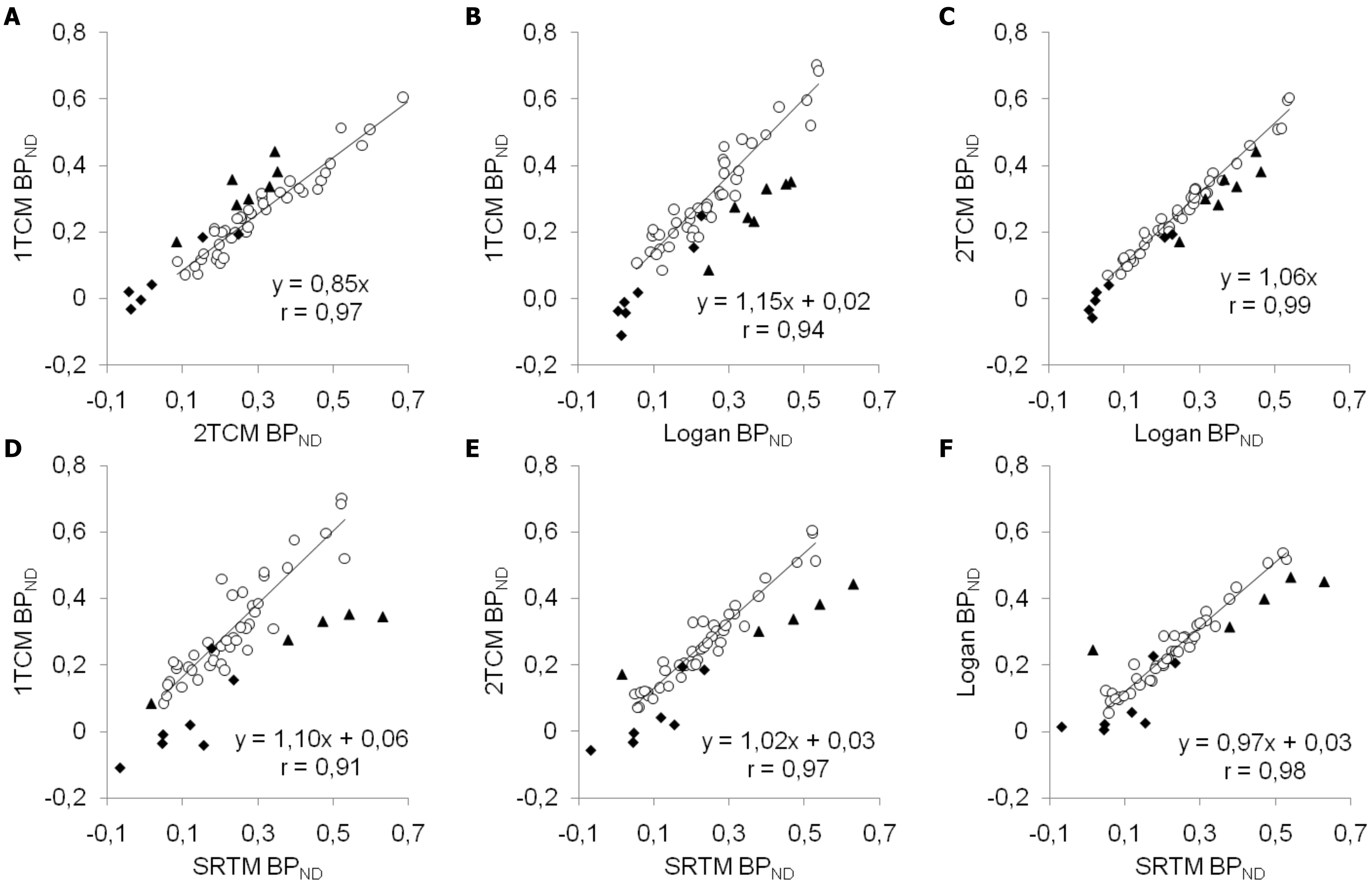
A
1TCM BP_{ND}
2TCM BP_{ND}
y = 0,85x
r = 0,97
B
1TCM BP_{ND}
Logan BP_{ND}
y = 1,15x + 0,02
r = 0,94
C
2TCM BP_{ND}
Logan BP_{ND}
y = 1,06x
r = 0,99
D
1TCM BP_{ND}
SRTM BP_{ND}
y = 1,10x + 0,06
r = 0,91
E
2TCM BP_{ND}
SRTM BP_{ND}
y = 1,02x + 0,03
r = 0,97
F
Logan BP_{ND}
SRTM BP_{ND}
y = 0,97x + 0,03
r = 0,98

Supplemental Figure 5. Mutual correlation of region-of-interest binding potential (BP_{ND}) values of [^{18}F]FEt-AMC13 obtained from different models. A – correlation of 1TCM and 2TCM BP_{ND} values; B,C – correlation of Logan-derived values with 1TCM and 2TCM-derived data; D,E,F – correlation of SRTM-derived values 1TCM, 2TCM and Logan-derived data. Linear regression lines, along with their equations and r and p values are shown on the graphs.

Points represent fits for individual regions in individual animals (both control and raclopride-pre-treated). Data for the striatum, hippocampus, thalamus, hypothalamus, cortex and brainstem are given as white circles. Data for the olfactory bulbs and pituitary are given as black diamonds and black triangles, respectively. These data are not used for the building of the linear regression lines shown on the graphs. BP_{ND} values are calculated using the cerebellum as a reference region, therefore the data for the cerebellum are not presented. V_T-based BP_{ND} values are used in case of 2TCM.

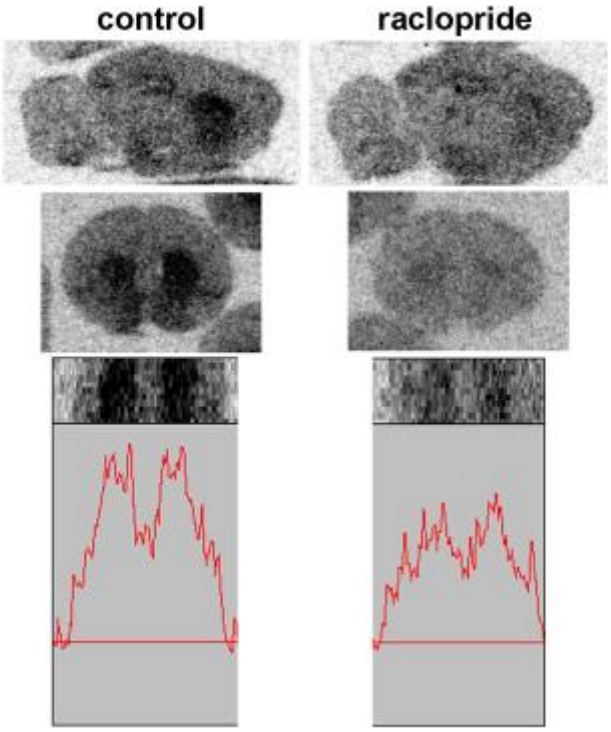

Supplemental Figure 6. Ex vivo autoradiography images of [^{18}F]FEt-AMC13 from control (left) and raclopride-treated (right) rats. Representative sagittal and coronal slices (top) and a profile section through the coronal slices showing absolute exposure values (bottom).

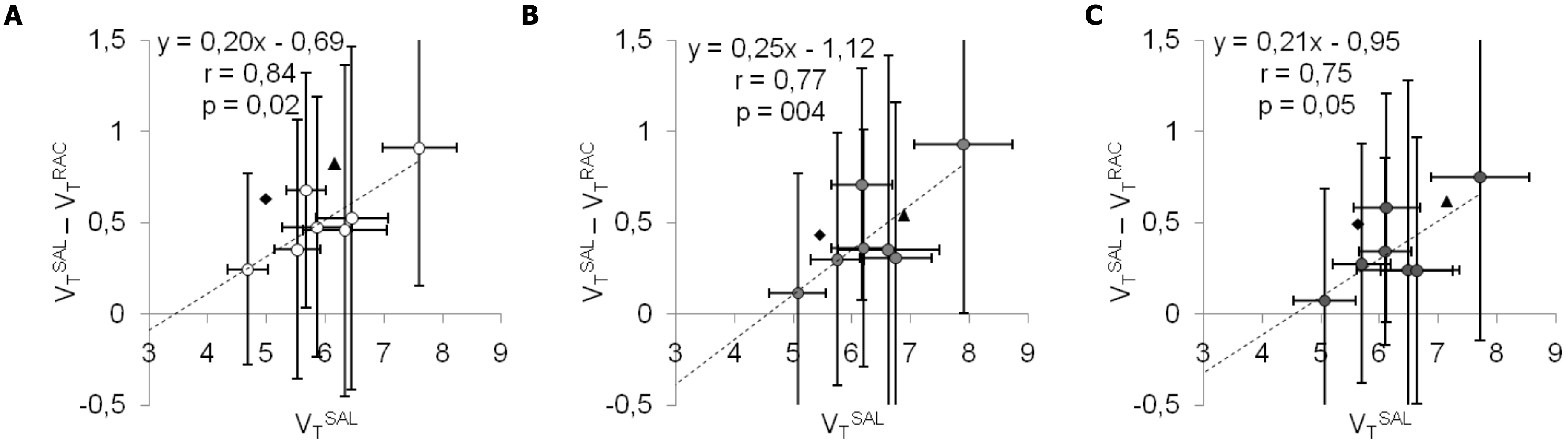

Supplemental Figure 7. Modified Lassen plots[2] for [^{18}F]FEt-AMC13 built from 1TCM (A), 2TCM (B) and Logan (C) data.

V_T^{SAL} –ROI distribution volume in the control group; V_T^{RAC} –ROI distribution volume in the raclopride-treated group. Assuming equal receptor occupancy by raclopride and equal non-displaceable volume of distribution (V_{ND}) of the tracer in all ROIs, the slope of the linear regression line represents the fraction of receptors occupied by raclopride, while the X-intersect represents V_{ND}.

Points show mean values, error bars show standard deviations. Data for striatum (always the rightmost topmost circle), hippocampus, thalamus, hypothalamus, cortex, brainstem and cerebellum (always the leftmost bottommost circle) are presented. Data for the olfactory bulbs and pituitary are given without error bars as, respectively, black diamonds and black triangles, and are not used for the building of the linear regression lines. Equations and r and p values of linear regression are shown on the graphs. Fits were performed disregarding the variability of the means.

SUPPLEMENTAL TABLES

Supplemental Table 1. Radio-HPLC and radio-TLC characterization data for [^{18}F]AMC13 and [^{18}F]AMC15 homologues.

Compound	QC1 k'	QC2 k’	TLC R_f
[^{18}F]FEt-AMC13	12.3*	2.1‡	0.40‖
[^{18}F]FPr-AMC13	12.9*	4.0‡	0.46‖
[^{18}F]FBu-AMC13	12.9*	5.9‡	0.56‖
[^{18}F]FEt-AMC15	9.1†	7.1§	0.16¶
[^{18}F]FPr-AMC15	9.8†	9.5§	0.22¶
[^{18}F]FBu-AMC15	12.2†	12.3§	0.50¶

*Platinum C18 EPS 100A 5 um, 250x5 mm; acetonitrile/water/formic acid 55/45/0.1; 2 ml/min

†Platinum C18 EPS 100A 5 um, 250x5 mm; acetonitrile/10 mM NaH_2PO_4 pH7.0 70/30; 2 ml/min

‡Gracesmart5 µm, 250x5 mm;acetonitrile/10 mM H_3PO_4 25/75, 2 ml/min

§Gracesmart5 µm, 250x5 mm;acetonitrile/10 mM NaH_2PO_4pH7.0 40/60, 2 ml/min

‖ethylacetate/methanol/triethylamine 100/5/1, silica plates

¶ethylacetate/methanol/ammonia/triethylamine 100/5/5/1, silica plates

Supplemental Table 2. MS/MS transitions and LC retention times of [^{18}F]AMC13 and [^{18}F]AMC15 homologues.

Compound	Precursor calcd M / formula	m/z $[M+H]^+$	Monitored transition products[a] calcd M / formula	m/z M^+	retention time, min
FEt-AMC13	331.16 $C_{19}H_{22}FNO_3$	332.20	153.07 $C_9H_{10}FO^+$<br>107.05 $C_7H_7O^+$	153.10<br>107.10	6.27
FPr-AMC13	345.17 $C_{20}H_{24}FNO_3$	346.20	167.09 $C_{10}H_{12}FO^+$<br>107.05 $C_7H_7O^+$	167.10<br>107.10	7.07
FBu-AMC13	359.19 $C_{21}H_{26}FNO_3$	360.20	181.10 $C_{11}H_{14}FO^+$<br>107.05 $C_7H_7O^+$	181.10<br>107.10	8.17
FEt-AMC15	429.24 $C_{24}H_{32}N_3O_3F$	430.30	206.12 $C_{12}H_{16}NO_2^+$	206.20	4.62
FPr-AMC15	443.26 $C_{25}H_{34}FN_3O_3$	444.30	206.12 $C_{12}H_{16}NO_2^+$	206.20	5.87
FBu-AMC15	457.27 $C_{26}H_{36}FN_3O_3$	458.30	206.12 $C_{12}H_{16}NO_2^+$	206.20	6.11

[a]Putative fragmentation reactions are as follows:

Q1 **Q3**

AMC13 homologues

n = 2: FEt-AMC13
n = 3: FPr-AMC13
n = 4: FBu-AMC13

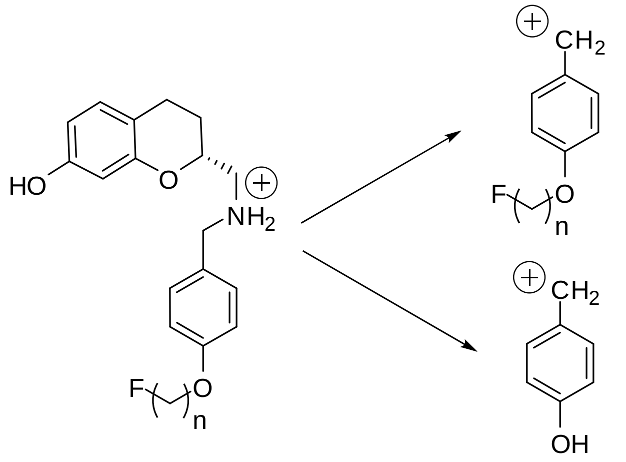

AMC15 homologues:

n = 2: FEt-AMC15
n = 3: FPr-AMC15
n = 4: FBu-AMC15

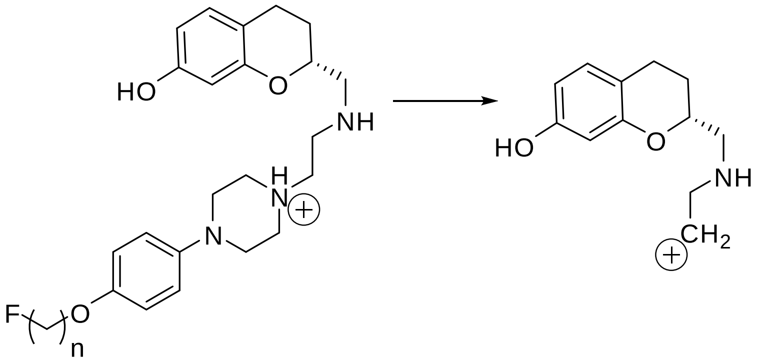

Supplemental Table 3. *Ex vivo* uptake(SUV) of the evaluated tracers in the peripheral tissues of saline (SAL) and raclopride-pre-treated (RAC) rats.

Tracer	[^{18}F]FEt-AMC13		[^{18}F]FBu-AMC13		[^{18}F]FEt-AMC15	
Treatment	SAL	RAC	SAL	RAC	SAL	RAC
Region	(n=4)	(n=4)	(n=3)	(n=3)	(n=3)	(n=3)
Whole blood	0.16±0.03	0.17±0.01	ND*	ND	0.06±0.03	0.12±0.08
Blood cells†	0.07±0.01	0.06±0.01	ND	ND	0.05±0.04	0.05±0.04
Plasma†	0.22±0.04	0.23±0.01	0.15±0.02	0.16±0.03	0.14±0.11	0.07±0.02
Adipose tissue	0.30±0.42	0.09±0.02	ND	ND	0.18±0.09	0.10±0.05
Adrenal glands	0.84±0.29	0.59±0.20	34.5±3.5	32.1±3.6	1.53±0.58	1.73±1.07
Bladder	1.00±0.52	0.83±0.64	0.43±0.16	0.59±0.26	0.23±0.05	0.69±0.30
Bone	0.24±0.21	0.17±0.05	0.73±0.47	0.77±0.46	0.14±0.03	0.12±0.08
Bone marrow	0.68±0.38	2.00±3.17	2.31±1.19	2.18±0.09	0.98±0.36	0.91±0.53
Caecum	0.46±0.29	0.63±0.84	ND	ND	0.15±0.10	0.91±1.10
Duodenum	1.15±0.25	4.01±4.63	3.24±1.2	2.70±1.37	1.92±0.35	22.8±36.6
Heart	0.15±0.04	0.16±0.02	0.50±0.25	0.36±0.03	0.11±0.04	0.15±0.10
Kidney	0.64±0.20	0.48±0.07	3.24±0.69	2.36±0.54	0.40±0.10	0.61±0.51
Large intestine	0.57±0.36	0.41±0.33	0.60±0.13	0.46±0.19	0.15±0.07	1.20±1.48
Large intestine content	0.06±0.03	0.09±0.12	ND	ND	0.02±0.01	0.06±0.04
Liver	1.51±0.14	1.43±0.30	1.49±0.15	1.99±0.86	0.86±0.23	1.47±1.56
Lung	1.67±0.41	1.38±0.20	4.85±2.15	4.26±1.02	1.18±0.16	1.19±0.40
Muscle	0.17±0.04	0.19±0.05	0.33±0.13	0.27±0.02	0.07±0.04	0.15±0.11
Pancreas	1.31±0.31	1.47±0.32	3.78±0.41	3.54±0.64	0.75±0.27	0.92±0.77
Prostate	0.62±0.18	0.40±0.26	0.95±0.17	1.27±0.40	0.38±0.15	0.42±0.10
Small intestine	3.15±2.00	2.39±1.94	4.52±5.94	3.21±3.67	0.18±0.07	11.1±18.4
Small intestine content	54.2±57.7	24.6±32.8	20.9±31.4	11.0±11.4	0.56±0.24	60.5±104
Spleen	0.46±0.05	0.52±0.10	2.48±1.27	2.14±0.20	1.52±0.68	1.62±1.15
Stomach	1.36±0.35	2.09±1.19	1.90±0.26	2.19±0.67	1.79±0.92	1.73±0.75
Submandibular gland	0.36±0.12	0.28±0.06	2.01±0.53	2.12±0.85	1.51±0.26	1.41±0.34
Testes	0.40±0.06	0.42±0.10	ND	ND	0.04±0.01	0.04±0.01
Thymus	0.32±0.06	0.30±0.06	2.04±0.27	1.54±0.12	0.49±0.09	0.43±0.15
Urine	11.0±9.5	4.40±3.88	0.63±0.53	1.51±0.95	0.69±0.33	2.32±3.35

Data are presented as means±SD * Not determined † Plasma and cell fractions were obtained from whole blood by short centrifugation (3500 g, 5 min)

Supplemental Table 4. 1TCM and 2TCM rate constant estimates of [^{18}F]FEt-AMC13 uptake in brain regions of control rats.

ROI	Rat	1TCM			2TCM				
		K_1 (ml/g/min)	k_2 (1/min)	AIC	K_1 (ml/g/min)	k_2 (1/min)	k_3 (1/min)	k_4 (1/min)	AIC
Striatum	A	0.45±0.03	0.05±0.01	489	1.77±0.62	4.95±3.00	1.34±0.29	0.05±0.01	449
	B	0.49±0.02	0.06±0.01	472	0.63±0.14	0.48±0.68	0.81±1.13	0.17±0.07	471
	C	0.47±0.02	0.07±0.004	456	0.98±0.85	3.47±8.55	2.81±3.01	0.11±0.08	454
	D	0.51±0.02	0.07±0.01	465	8.06±30.34	58.0±203.8	3.54±188.40	0.07±263.98	433
Hippocampus	A	0.41±0.02	0.06±0.01	483	2.21±1.25	8.56±3.62	1.60±2.24	0.05±8.88	443
	B	0.51±0.02	0.08±0.01	466	0.71±0.07	0.50±0.23	0.50±0.27	0.14±0.03	449
	C	0.46±0.01	0.08±0.004	441	0.60±0.12	0.57±0.77	1.17±1.34	0.24±0.09	438
	D	0.48±0.02	0.08±0.01	455	101±14615	1246±181990	5.49±4.23	0.07±0.05	441
Thalamus	A	0.44±0.02	0.06±0.01	475	14.0±117.5	104±848	3.05±820.48	0.05±961.03	452
	B	0.65±0.03	0.10±0.01	473	0.88±0.12	0.58±0.58	0.67±1.63	0.19±1.99	464
	C	0.55±0.02	0.09±0.01	460	0.71±0.13	0.48±0.97	0.76±3.17	0.23±3.61	457
	D	0.64±0.03	0.11±0.01	479	3.13±3.18	12.3±18.9	2.61±0.96	0.11±0.02	453
Hypothalamus	A	0.40±0.03	0.06±0.01	493	273*	1355*	1.65*	0.05*	459
	B	0.53±0.03	0.09±0.01	478	1.17±0.88	3.45±1.30	2.22±6.73	0.13±14.19	472
	C	0.50±0.02	0.08±0.01	460	0.64±0.24	0.63±1.60	1.37±2.85	0.27±0.20	462
	D	0.59±0.04	0.12±0.02	489	680±116990	3105±534810	2.16±0.48	0.09±0.01	453
Cortex	A	0.37±0.02	0.06±0.01	468	1.68±0.46	7.83±1.43	1.84±0.62	0.06±4.47	401
	B	0.47±0.02	0.09±0.01	450	0.66±0.04	0.64±0.21	0.72±0.24	0.16±0.02	416
	C	0.41±0.01	0.07±0.002	417	0.43±0.12	0.19±1.58	1.50±19.49	1.00±1.64	421
	D	0.47±0.02	0.09±0.01	452	1.46±0.45	6.65±0.77	2.62±1.18	0.11±6.33	403
Brainstem	A	0.63±0.04	0.10±0.02	501	2.03±0.40	3.53±1.32	1.05±0.19	0.10±0.01	451
	B	0.60±0.03	0.11±0.01	475	0.96±0.11	0.89±0.30	0.72±0.83	0.15±1.20	449
	C	0.68±0.02	0.12±0.01	467	1.11±0.22	1.19±0.59	1.10±1.86	0.21±2.66	452
	D	0.71±0.03	0.13±0.01	479	4.75±5.87	19.6±19.9	2.79±15.04	0.12±37.81	446
Cerebellum	A	0.57±0.04	0.11±0.02	492	2.10±0.32	4.90±1.30	1.23±0.13	0.10±0.005	420
	B	0.61±0.02	0.13±0.01	461	0.81±0.05	0.53±0.24	0.46±0.55	0.20±0.71	436
	C	0.62±0.01	0.13±0.01	439	0.86±0.21	1.13±1.45	1.84±1.78	0.34±0.12	433
	D	0.68±0.03	0.16±0.02	473	48.7±819.7	298±5152	3.55±1.05	0.13±0.03	438

(continued on the next page)

Supplemental Table 4 (continuation). 1TCM and 2TCM rate constant estimates of [^{18}F]FEt-AMC13 uptake in brain regions of control rats.

ROI	Rat	1TCM			2TCM				
		K_1 (ml/g/min)	k_2 (1/min)	AIC	K_1 (ml/g/min)	k_2 (1/min)	k_3 (1/min)	k_4 (1/min)	AIC
Olfactory bulbs	A	0.55±0.05	0.12±0.02	500	3.58±1.65	9.75±3.75	1.20±2.96	0.09±7.03	449
	B	0.49±0.02	0.08±0.01	453	0.63±0.07	0.47±0.34	0.69±0.56	0.20±0.04	444
	C	0.44±0.01	0.09±0.005	438	0.59±0.10	0.68±0.93	1.07±3.21	0.23±3.75	431
	D	0.50±0.03	0.10±0.01	479	1.08±0.20	1.82±0.90	0.92±0.31	0.11±0.01	452
Pituitary	A	0.52±0.05	0.08±0.02	510	6.16±6.57	16.8±16.5	1.11±15.04	0.06±22.82	472
	B	0.63±0.04	0.10±0.01	490	1.23±0.31	1.62±0.42	0.99±1.42	0.12±2.70	474
	C	0.52±0.02	0.08±0.01	472	0.97±0.34	1.54±0.73	1.23±2.61	0.13±4.28	464
	D	0.67±0.05	0.12±0.02	497	1.38±0.24	1.48±0.20	0.69±0.58	0.11±1.34	473

Data are presented as means±SD.* Standard deviation could not be estimated

Supplemental Table 5. V_T of [^{18}F]FEt-AMC13 per brain region in control and raclopride-treated rats.

Region of interest	Logan V_T		1TCM V_T		2TCM V_T	
	control	raclopride	control	raclopride	control	raclopride
Striatum	7.72±0.84 (11)	6.97±0.32 (5)	7.60±0.64 (8)	6.69±0.41 (6)	7.90±0.84 (11)	6.97±0.39 (6)
Hippocampus	6.49±0.88 (14)	6.25±0.56 (9)	6.34±0.71 (11)	5.88±0.56 (10)	6.62±0.86 (13)	6.27±0.63 (10)
Thalamus	6.64±0.61 (9)	6.40±0.40 (6)	6.45±0.62 (10)	5.93±0.71 (12)	6.75±0.62 (9)	6.44±0.60 (9)
Hypothalamus	6.10±0.44 (7)	5.76±0.26 (5)	5.86±0.60 (10)	5.38±0.38 (7)	6.20±0.55 (9)	5.83±0.34 (6)
Cortex	5.69±0.49 (9)	5.42±0.44 (8)	5.53±0.38 (7)	5.17±0.60 (12)	5.75±0.45 (8)	5.45±0.52 (10)
Brainstem	6.12±0.57 (9)	5.53±0.26 (5)	5.67±0.33 (6)	4.99±0.56 (11)	6.17±0.52 (8)	5.46±0.36 (7)
Cerebellum	5.06±0.53 (11)	4.99±0.30 (6)	4.68±0.34 (7)	4.43±0.40 (9)	5.08±0.49 (10)	4.96±0.44 (9)
Olfactory Bulbs	5.64±0.57 (10)	5.14±0.43 (8)	4.99±0.62 (12)	4.36±0.55 (13)	5.45±0.52 (10)	5.02±0.61 (12)
Pituitary	7.15±0.49 (7)	6.53±0.44 (7)	6.16±0.35 (6)	5.33±0.88 (17)	6.89±0.32 (5)	6.34±0.82 (13)

Data are presented as means±SD, n=4 for the control group, n=3 for the raclopride-treated group. Coefficients of variation (in %) are shown in parentheses.

Supplemental Table 6. BP_{ND} of [^{18}F]FEt-AMC13 per brain region in control and raclopride-treated rats.

Region of interest	1TCM BP_{ND}		2TCM BP_{ND} (V_T-based)		Logan BP_{ND}		SRTM BP_{ND}	
	control	raclopride	control	raclopride	control	raclopride	control	raclopride
Striatum	0.63±0.08	0.51±0.06 (-18%)	0.56±0.05	0.41±0.05 (-27%)*	0.52±0.01	0.40±0.04 (-24%)*	0.51±0.02	0.38±0.05 (–26%)**
Hippocampus	0.35±0.11	0.33±0.09 (-8%)	0.30±0.07	0.26±0.06 (-13%)	0.28±0.04	0.25±0.05 (-10%)	0.26±0.04	0.23±0.05 (–13%)
Thalamus	0.38±0.06	0.33±0.04 (-12%)	0.33±0.02	0.30±0.01 (-10%) *	0.31±0.02	0.28±0.005 (-10%)*	0.28±0.06	0.26±0.02 (–7%)
Hypothalamus	0.25±0.04	0.22±0.04 (-14%)	0.22±0.03	0.18±0.06 (-19%)	0.21±0.04	0.16±0.04 (-25%)	0.21±0.03	0.16±0.04 (–23%)
Cortex	0.18±0.05	0.17±0.05 (-9%)	0.13±0.04	0.10±0.02 (-27%)	0.13±0.03	0.09±0.03 (-32%)	0.11±0.03	0.08±0.02 (–24%)
Brainstem	0.21±0.03	0.13±0.03 (-41%)*	0.22±0.02	0.10±0.02 (-53%)**	0.21±0.02	0.11±0.02 (-47%)**	0.18±0.04	0.07±0.03 (–60%)**
Olfactory Bulbs	0.07±0.16	-0.02±0.03 (-127%)	0.08±0.13	0.01±0.04 (-88%)	0.12±0.12	0.03±0.03 (-74%)	0.10±0.13	0.11±0.05 (+10%)
Pituitary	0.32±0.05	0.18±0.05 (-38%)	0.36±0.07	0.13±0.04 (-23%)	0.42±0.05	0.13±0.03 (-26%)	0.71±0.32	0.55±0.53 (–22%)

Data are presented as means±SD, n=4 for the control group, n=3 for the raclopride-treated group (except for the SRTM BP_{ND} values, where n=4).

Next to the BP_{ND} values of the raclopride-treated group, percentage of change relative to the control group is shown. * $P < 0.05$, ** $P < 0.01$, 2-sided Welch test

LITERATURE CITED

1. Innis RB, Cunningham VJ, Delforge J, et al. Consensus nomenclature for in vivo imaging of reversibly binding radioligands. J Cereb Blood Flow Metab. 2007;27(9):1533–9.

2. Cunningham VJ, Rabiner EA, Slifstein M, Laruelle M, Gunn RN. Measuring drug occupancy in the absence of a reference region: the Lassen plot re-visited. J Cereb Blood Flow & Metab. 2009;30(1):46–50.
